# Random Thoughts

**Published:** 1856-12

**Authors:** 


### RANDOM THOUGHTS,

*Suggested by a hasty reading of the Proceedings of the American Dental Convention.*

The first thought that strays into the inclosure of our imagination is the great advantages derived from combined effort. It will be seen that the association got itself organized and ready for business, in the short space of *five hours* from the time the president took the chair. Now, no one would be silly enough to believe that this could have been done, had not a few of the most kind-hearted and able-bodied members zealously went to work, "moving," seconding, offering amendments, appealing, making long speeches, etc.; evincing a full consciousness of the fact that the thing must be organized, and none but they could do it. With less zeal and harmony, indeed, it is not probable that even they would have succeeded. Had it been permitted to fall together, and go at once into action, the whole affair might have been destroyed by the concussion. Had it been delayed *much* beyond the five hours, some might have become impatient, and left in disgust. Each fragment was *held back* till just the exact time, and then quietly slid into its proper niche. We trust it can be "put through" without such mighty efforts next year. If it can not, it is to be hoped the same force will be on hand, lest raw recruits might fail.

The next thought that presents itself is the active and earnest part taken in the practical discussions by the older and more experienced members of the profession. "Age is honorable"; and this time it has shown itself peculiarly so. We only regret that we younger members are apt to forget to show them that deference to which they are entitled. But, then, they are so good-natured, they forgive us as fast as we transgress. It is unfortunate that the report so often fails to do justice to their remarks; though it must be expected that, in the use of technicalities, reporters will be at a loss sometimes. But what was the committee doing so long? Could they not correct some of the most glaring mistakes? Did Dr. Taylor say, "The chemical agent which acted on the *cartilaginous* parts was certainly different from that which acted on the *living* parts?" Did Dr. Arthur speak of a *mechanical* view respecting the action of escharotics on dentine? Did Dr. Harris convey the idea that but four acids are capable of acting chemically on the teeth? or that no acid acts *chemically* on the teeth unless it *decomposes* the phosphate of lime?

Another thought is the beautiful and professional (?) application of the "self-puff and reciprocal tickle." It reminds us of the discussions of the "vegetable astringent question," at a meeting of the "FOREST ASSOCIATION."

"DR. BLACKBERRY said that in regard to astringency, he counted that he could do his own drawing, that perhaps he needed as little aid in that way as others,—in short, that he considered himself *some* on a *pucker*. He could contract the lips of a blooming lass so tightly, that a five-pound pressure, in the shape of a lover's kiss, would fail to produce even the slightest 'smack;' and any member of the Forest Association, by the use of the same skill, talent, and perseverance, can do the same. He obtained his first hints in this direction from Dr. PERSIMMON; but he had learned that the Doctor, in his *maturity*, discards the astringent practice, *in toto*, and disclaims that he ever practiced it to any extent. He could not say that he could do all with *brier-root* that could be done with other agents. An achievement of Dr. Whiteoak with nutgall and tannin, induced him to suspect he could not. He had witnessed an operation in which Dr. W. had, by a single application to the mouth of a spendthrift's purse, saved a handsome balance of a partially spent patrimony.

Dr. POPLAR said that no doubt tannin and galls are astringent; so is extract of brier-root. A year and a half ago he had applied it to two double harelips, and the patients had been whistling "Yankee Doodle" ever since. Nor was this a remarkable case. The mastiff of his friend Dr. Dogwood had an attack of hydrophobia. The extract prepared by Dr. Blackberry himself, was injected into the mouth, which, by contraction of the mucous membrane, became perfectly closed, rendering him entirely harmless.

Dr. MOUNTAINASH said that he always thought he had some astringent abilities; but having witnessed an important operation by Dr. Whiteoak, which was the application of *gall* to a rich man's heart, and seeing that it contracted so close as to leave no room for even the smallest tender emotion, he could make no further pretensions.

Dr. CEDAR said that he rose to give the experience of others rather than his own. Doubts had been expressed in regard to gall and tannin; but they were groundless. A case just now occurred to him of a politician, whose conscience, if he was not mistaken, was entirely gone, except a slight sensibility in regard to laboring for his party without pay, being perfectly restored to the paths of rectitude, and fitted for ordinary pursuits. His case was managed by my young friend, Dr. SPRUCEPINE, of this grove."

As this discussion took place many years ago, it is interesting to notice the results. The debate was extensively and minutely reported on the foliage of the surrounding groves, and was extensively read by the feathered tribes. As a matter of course, they concluded the professional trees so lauded *must* be heroes; and, therefore, they would regale themselves in their shadows, sing among their branches, and rear their young under their protecting foliage. Hence, all Fence Row is alive to this day with the friends and patrons of Dr. Blackberry; Dr. Sprucepine bends beneath the weight of his feathered friends; Dr. Poplar's office became at once the fashionable resort of high-flying birds; and Dr. Whiteoak, whose modesty was so severely tested by the puffs of his astringent achievements, is by no means left in silent loneliness.

Now, whether the success of our dental brethren will be commensurate with that of their forest friends, from whom they have, probably, taken the hint, remains to be seen. If it should, it is probable the process can be extended by the next meeting.

Another thought, pertaining to festivities, suggests itself. In the event of a social entertainment at a future meeting, would it not be well to erect a power grindstone, for the accommodation of those who have axes to grind,—no matter whether the axes be used for the work of a private office, or a *public institution?* When an ax has been badly roughened by the quarrels of rival choppers, it grinds harshly on the feelings of a social assembly.

But the prominent thought that suggests itself, first, last, and all the time, is, that the Convention was a great and glorious success. It was the assembly of assemblies. The report of its discussions can not fail to do much in the cause of dental advancement. Even its existence is a proof of the already advanced state of the profession. And the largely increased attendance at the second annual meeting is a striking evidence of progressive energy. BUCKEYE.

